# [μ-1,3-Bis(diphenyl­phosphino)propane-κ^2^ *P*:*P*′]bis­[bromidogold(I)]

**DOI:** 10.1107/S1600536810001406

**Published:** 2010-01-16

**Authors:** Fabian Mohr, Anja Molter, Edward R. T. Tiekink

**Affiliations:** a Fachbereich C – Anorganische Chemie, Bergische Universität Wuppertal, 42119 Wuppertal, Germany; b Department of Chemistry, University of Malaya, 50603 Kuala Lumpur, Malaysia

## Abstract

The title compound, [Au_2_Br_2_(C_27_H_26_P_2_)], features linearly coordinated Au^I^ atoms within *P*,*Br*-donor sets. The central portion of the mol­ecule is practically planar as quanti­fied by the Br–Au⋯Au–Br torsion angle of −169.9 (2)°. The P—Au—Br chromophores are twisted with respect to each other [dihedral angle = 52.3 (6)°]. The benzene rings on each P atom lie on either side of this plane. The Au atoms are positioned at the periphery of the mol­ecule, which facilitates the formation of Au⋯Au inter­actions [3.2575 (11) Å] that result in the formation of supra­molecular chains along the *b*-axis direction. The Au⋯Au inter­actions are responsible for the deviations from the ideal linear geometry for each Au atom.

## Related literature

For polymorphic structures of the chlorido analogue of the title compound, see: Cooper *et al.* (1984 ▶); Kaim *et al.* (2005 ▶). For background to related studies in gold chemistry, see: Gallenkamp *et al.* (2009 ▶).
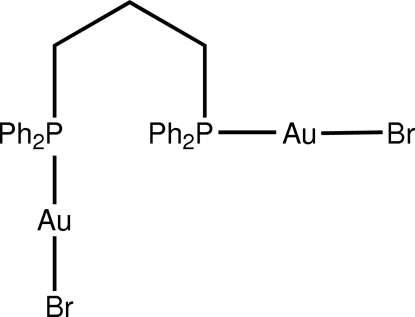


## Experimental

### 

#### Crystal data

[Au_2_Br_2_(C_27_H_26_P_2_)]
*M* *_r_* = 966.17
Orthorhombic, 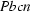

*a* = 19.610 (5) Å
*b* = 14.322 (4) Å
*c* = 19.958 (5) Å
*V* = 5605 (2) Å^3^
*Z* = 8
Mo *K*α radiation
μ = 13.44 mm^−1^
*T* = 98 K
0.35 × 0.09 × 0.04 mm

#### Data collection

Rigaku AFC12/SATURN724 diffractometer
Absorption correction: multi-scan (*ABSCOR*; Higashi, 1995 ▶) *T*_min_ = 0.355, *T*_max_ = 1
33240 measured reflections
5794 independent reflections
5470 reflections with *I* > 2σ(*I*)
*R*_int_ = 0.073

#### Refinement

*R*[*F* ^2^ > 2σ(*F* ^2^)] = 0.058
*wR*(*F* ^2^) = 0.139
*S* = 1.23
5794 reflections
298 parameters
H-atom parameters constrained
Δρ_max_ = 2.34 e Å^−3^
Δρ_min_ = −2.69 e Å^−3^

### 

Data collection: *CrystalClear* (Rigaku/MSC, 2005 ▶); cell refinement: *CrystalClear*; data reduction: *CrystalClear*; program(s) used to solve structure: *SHELXS97* (Sheldrick, 2008 ▶); program(s) used to refine structure: *SHELXL97* (Sheldrick, 2008 ▶); molecular graphics: *ORTEP-3* (Farrugia, 1997 ▶) and *DIAMOND* (Brandenburg, 2006 ▶); software used to prepare material for publication: *publCIF* (Westrip, 2010 ▶).

## Supplementary Material

Crystal structure: contains datablocks global, I. DOI: 10.1107/S1600536810001406/hg2623sup1.cif

Structure factors: contains datablocks I. DOI: 10.1107/S1600536810001406/hg2623Isup2.hkl

Additional supplementary materials:  crystallographic information; 3D view; checkCIF report

## Figures and Tables

**Table:** 

Au1—Br1	2.4128 (13)
Au1—P1	2.246 (3)
Au2—Br2	2.4170 (12)
Au2—P2	2.258 (3)

**Table:** 

P1—Au1—Br1	171.73 (7)
P2—Au2—Br2	174.31 (8)

**Fig. 1.**
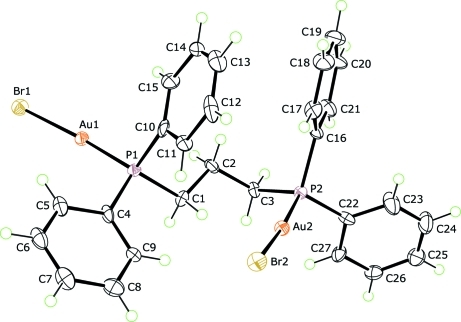
Molecular structure of (I), showing atom-labelling scheme and displacement ellipsoids at the 50% probability level.

**Fig. 2.**
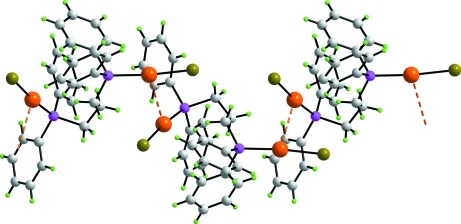
A view of the supramolecular chain in (I) mediated by Au···Au interactions (orange dashed lines). Color code: Au, orange; Br, olive; P, pink; C, grey; and H, green.

## supplementary crystallographic information

### Comment

The title compound (I) was prepared as a precursor material during studies into the biological activity of phosphinegold(I) thiolates (Gallenkamp *et al.*, 2009). The molecular structure of (I), Fig. 1, features two linearly coordinated Au atoms defined by P and Br donor atoms, Table 1. The pairs of Au–Br and Au–P bond distances are equal within experimental error, Table 1. The central part of the molecule is approximately planar as quantified by the torsion angle Br1–Au1···Au2–Br2 of -169.91 (21) °. The propylene bridge and phosphorus atoms lie in this plane with the two benzene rings, one from each phosphorus atom, above and below the plane. The P–Au–Br chromophores are approximately orthogonal to each other. The deviations from the ideal linear geometries about the gold atoms are likely to arise from the formation of intermolecular Au···Au interactions. Each of the gold atoms lies external to but on different sides of the molecule to facilitate the formation of aurophilic, Au···Au, interactions [Au1···Au2^i^ = 3.2575 (11) Å for i: 1/2 - *x*, -1/2 + *y*, *z*]. These interactions result in the formation of a supramolecular chain along the *b* axis, Fig. 2, and are likely responsible for the distortions from the ideal linear geometries for the gold atoms, Table 1.

Compound (I) is isomorphous with the chloro analogue (Cooper *et al.*, 1984) for which the intermolecular Au···Au distance was 3.316 (9) Å. A second polymorph of the chloro derivative is known which adopts a *closo* structure with an intramolecular Au···Au interaction of 3.2368 (9) Å (Kaim *et al.*, 2005).

### Experimental

Crystals of (dppp)Au_2_Br_2_ were isolated from an attempted reaction of (dppp)Au_2_Br_2_ with a selenourea ligand in the presence of a base in CH_2_2Cl_2_ solution (Gallenkamp *et al.*,  2009).

### Refinement

The C-bound H atoms were geometrically placed (C–H = 0.95–0.99 Å) and refined as riding with *U*_iso_(H) = 1.2*U*_eq_(C). The maximum and minimum residual electron density peaks of 2.34 and -2.69 e Å^-3^, respectively, were located 1.13 Å and 0.95 Å from the Au1 atom.

### Crystal data

**Table:**

[Au_2_Br_2_(C_27_H_26_P_2_)]	*F*(000) = 3568
*M**_r_* = 966.17	*D*_x_ = 2.290 Mg m^−^^3^
Orthorhombic, *P**b**c**n*	Mo *K*α radiation, λ = 0.71070 Å
Hall symbol: -P 2n 2ab	Cell parameters from 32332 reflections
*a* = 19.610 (5) Å	θ = 2.0–40.7°
*b* = 14.322 (4) Å	µ = 13.44 mm^−^^1^
*c* = 19.958 (5) Å	*T* = 98 K
*V* = 5605 (2) Å^3^	Plate, light-brown
*Z* = 8	0.35 × 0.09 × 0.04 mm

### Data collection

**Table:**

Rigaku AFC12K/SATURN724 diffractometer	5794 independent reflections
Radiation source: fine-focus sealed tube	5470 reflections with *I* > 2σ(*I*)
graphite	*R*_int_ = 0.073
ω scans	θ_max_ = 26.5°, θ_min_ = 1.8°
Absorption correction: multi-scan (*ABSCOR*; Higashi, 1995)	*h* = −24→24
*T*_min_ = 0.355, *T*_max_ = 1	*k* = −17→17
33240 measured reflections	*l* = −25→20

### Refinement

**Table:**

Refinement on *F*^2^	Primary atom site location: structure-invariant direct methods
Least-squares matrix: full	Secondary atom site location: difference Fourier map
*R*[*F*^2^ > 2σ(*F*^2^)] = 0.058	Hydrogen site location: inferred from neighbouring sites
*wR*(*F*^2^) = 0.139	H-atom parameters constrained
*S* = 1.23	*w* = 1/[σ^2^(*F*_o_^2^) + (0.0548*P*)^2^ + 72.0449*P*] where *P* = (*F*_o_^2^ + 2*F*_c_^2^)/3
5794 reflections	(Δ/σ)_max_ = 0.001
298 parameters	Δρ_max_ = 2.34 e Å^−^^3^
0 restraints	Δρ_min_ = −2.69 e Å^−^^3^

### Special details

**Table:**

Geometry. All s.u.'s (except the s.u. in the dihedral angle between two l.s. planes) are estimated using the full covariance matrix. The cell s.u.'s are taken into account individually in the estimation of s.u.'s in distances, angles and torsion angles; correlations between s.u.'s in cell parameters are only used when they are defined by crystal symmetry. An approximate (isotropic) treatment of cell s.u.'s is used for estimating s.u.'s involving l.s. planes.
Refinement. Refinement of *F*^2^ against ALL reflections. The weighted *R*-factor *wR* and goodness of fit *S* are based on *F*^2^, conventional *R*-factors *R* are based on *F*, with *F* set to zero for negative *F*^2^. The threshold expression of *F*^2^ > 2σ(*F*^2^) is used only for calculating *R*-factors(gt) *etc*. and is not relevant to the choice of reflections for refinement. *R*-factors based on *F*^2^ are statistically about twice as large as those based on *F*, and *R*- factors based on ALL data will be even larger.

### Fractional atomic coordinates and isotropic or equivalent isotropic displacement parameters (Å^2^)

**Table:**

	*x*	*y*	*z*	*U*_iso_*/*U*_eq_	
Au1	0.14604 (2)	0.53723 (3)	0.620369 (19)	0.01793 (13)	
Au2	0.21598 (2)	0.98204 (3)	0.538567 (18)	0.01725 (13)	
Br1	0.13248 (6)	0.37156 (7)	0.63780 (5)	0.0240 (2)	
Br2	0.15705 (6)	1.05983 (8)	0.62910 (5)	0.0257 (3)	
P1	0.14836 (14)	0.69395 (18)	0.61668 (12)	0.0166 (5)	
P2	0.26129 (14)	0.90170 (19)	0.45196 (12)	0.0160 (5)	
C1	0.2237 (6)	0.7464 (7)	0.5797 (5)	0.018 (2)	
H1A	0.2210	0.8150	0.5853	0.021*	
H1B	0.2644	0.7243	0.6043	0.021*	
C2	0.2331 (6)	0.7243 (7)	0.5047 (5)	0.020 (2)	
H2A	0.2445	0.6574	0.4993	0.024*	
H2B	0.1898	0.7364	0.4807	0.024*	
C3	0.2900 (6)	0.7840 (7)	0.4738 (5)	0.017 (2)	
H3A	0.3072	0.7527	0.4329	0.021*	
H3B	0.3283	0.7886	0.5059	0.021*	
C4	0.1402 (6)	0.7438 (8)	0.6997 (5)	0.020 (2)	
C5	0.0974 (6)	0.6998 (8)	0.7471 (5)	0.025 (2)	
H5	0.0774	0.6411	0.7371	0.030*	
C6	0.0845 (6)	0.7424 (9)	0.8087 (5)	0.030 (3)	
H6	0.0560	0.7119	0.8404	0.036*	
C7	0.1123 (6)	0.8278 (8)	0.8241 (5)	0.028 (3)	
H7	0.1017	0.8571	0.8655	0.034*	
C8	0.1573 (7)	0.8723 (9)	0.7779 (6)	0.029 (3)	
H8	0.1782	0.9300	0.7892	0.035*	
C9	0.1704 (6)	0.8308 (7)	0.7165 (5)	0.022 (2)	
H9	0.1998	0.8608	0.6854	0.026*	
C10	0.0788 (6)	0.7460 (8)	0.5690 (5)	0.023 (2)	
C11	0.0562 (5)	0.8379 (8)	0.5832 (5)	0.021 (2)	
H11	0.0748	0.8717	0.6198	0.025*	
C12	0.0060 (7)	0.8779 (9)	0.5424 (6)	0.036 (3)	
H12	−0.0098	0.9392	0.5520	0.043*	
C13	−0.0212 (6)	0.8300 (9)	0.4881 (6)	0.032 (3)	
H13	−0.0556	0.8578	0.4613	0.039*	
C14	0.0028 (7)	0.7409 (9)	0.4736 (6)	0.033 (3)	
H14	−0.0148	0.7086	0.4358	0.039*	
C15	0.0514 (6)	0.6983 (8)	0.5129 (5)	0.026 (2)	
H15	0.0665	0.6370	0.5025	0.031*	
C16	0.1996 (5)	0.8807 (7)	0.3852 (5)	0.017 (2)	
C17	0.1306 (6)	0.9081 (9)	0.3939 (6)	0.028 (3)	
H17	0.1167	0.9401	0.4333	0.033*	
C18	0.0837 (7)	0.8871 (9)	0.3435 (6)	0.032 (3)	
H18	0.0377	0.9067	0.3482	0.038*	
C19	0.1032 (7)	0.8381 (9)	0.2867 (5)	0.033 (3)	
H19	0.0704	0.8230	0.2534	0.039*	
C20	0.1712 (7)	0.8111 (9)	0.2783 (5)	0.028 (3)	
H20	0.1846	0.7782	0.2392	0.034*	
C21	0.2197 (6)	0.8326 (9)	0.3281 (5)	0.026 (2)	
H21	0.2659	0.8142	0.3226	0.032*	
C22	0.3332 (6)	0.9573 (8)	0.4126 (5)	0.021 (2)	
C23	0.3253 (8)	1.0161 (11)	0.3567 (7)	0.044 (4)	
H23	0.2809	1.0268	0.3393	0.053*	
C24	0.3804 (8)	1.0586 (10)	0.3266 (7)	0.040 (3)	
H24	0.3737	1.0967	0.2882	0.049*	
C25	0.4445 (7)	1.0462 (9)	0.3514 (6)	0.032 (3)	
H25	0.4822	1.0758	0.3305	0.038*	
C26	0.4542 (6)	0.9906 (8)	0.4069 (6)	0.027 (3)	
H26	0.4988	0.9837	0.4249	0.033*	
C27	0.4003 (6)	0.9448 (8)	0.4369 (6)	0.024 (2)	
H27	0.4084	0.9048	0.4740	0.029*	

### Atomic displacement parameters (Å^2^)

**Table:**

	*U*^11^	*U*^22^	*U*^33^	*U*^12^	*U*^13^	*U*^23^
Au1	0.0139 (2)	0.0208 (2)	0.0191 (2)	−0.00115 (14)	−0.00109 (14)	0.00287 (14)
Au2	0.0146 (2)	0.0192 (2)	0.0179 (2)	0.00048 (15)	0.00127 (14)	0.00027 (13)
Br1	0.0204 (6)	0.0247 (5)	0.0271 (5)	0.0012 (4)	−0.0007 (4)	0.0003 (4)
Br2	0.0236 (6)	0.0273 (5)	0.0261 (5)	0.0012 (4)	0.0050 (4)	−0.0038 (4)
P1	0.0129 (14)	0.0184 (12)	0.0186 (12)	−0.0024 (10)	−0.0020 (10)	0.0024 (9)
P2	0.0102 (13)	0.0218 (13)	0.0160 (11)	−0.0023 (10)	0.0021 (9)	0.0014 (9)
C1	0.017 (6)	0.014 (4)	0.022 (5)	0.000 (4)	0.003 (4)	0.002 (4)
C2	0.023 (6)	0.026 (5)	0.012 (4)	0.000 (4)	−0.003 (4)	0.001 (4)
C3	0.017 (6)	0.019 (5)	0.015 (4)	0.003 (4)	0.003 (4)	0.005 (4)
C4	0.012 (6)	0.033 (6)	0.015 (4)	0.001 (4)	0.005 (4)	0.009 (4)
C5	0.025 (7)	0.029 (6)	0.021 (5)	0.003 (5)	0.002 (5)	0.008 (4)
C6	0.021 (7)	0.048 (7)	0.020 (5)	0.004 (5)	0.004 (4)	0.010 (5)
C7	0.026 (7)	0.037 (6)	0.021 (5)	0.011 (5)	0.007 (5)	0.000 (4)
C8	0.024 (7)	0.035 (6)	0.028 (5)	0.004 (5)	−0.005 (5)	−0.001 (5)
C9	0.025 (6)	0.026 (5)	0.014 (4)	−0.005 (4)	−0.004 (4)	0.002 (4)
C10	0.013 (6)	0.028 (6)	0.029 (5)	−0.007 (4)	0.003 (4)	0.015 (4)
C11	0.008 (5)	0.034 (6)	0.021 (5)	0.000 (4)	0.000 (4)	0.005 (4)
C12	0.034 (8)	0.040 (7)	0.034 (6)	0.010 (6)	0.008 (5)	0.022 (5)
C13	0.013 (6)	0.051 (8)	0.033 (6)	0.006 (5)	0.000 (5)	0.016 (5)
C14	0.028 (8)	0.044 (7)	0.025 (5)	−0.019 (6)	−0.012 (5)	0.014 (5)
C15	0.012 (6)	0.038 (6)	0.027 (5)	−0.003 (5)	0.000 (4)	0.002 (5)
C16	0.011 (5)	0.027 (5)	0.014 (4)	−0.001 (4)	−0.001 (4)	0.005 (4)
C17	0.009 (6)	0.043 (7)	0.031 (6)	−0.004 (5)	0.006 (4)	0.008 (5)
C18	0.014 (6)	0.048 (7)	0.034 (6)	0.000 (5)	−0.001 (5)	0.002 (5)
C19	0.029 (7)	0.044 (7)	0.025 (5)	−0.005 (6)	−0.009 (5)	−0.001 (5)
C20	0.029 (7)	0.041 (7)	0.015 (5)	0.000 (5)	−0.004 (5)	−0.006 (4)
C21	0.021 (7)	0.040 (7)	0.019 (5)	0.003 (5)	0.001 (4)	0.005 (4)
C22	0.019 (6)	0.029 (6)	0.015 (5)	0.003 (4)	−0.006 (4)	−0.002 (4)
C23	0.019 (8)	0.067 (10)	0.046 (8)	−0.004 (7)	−0.008 (6)	0.032 (7)
C24	0.032 (8)	0.048 (8)	0.042 (7)	−0.009 (6)	0.000 (6)	0.024 (6)
C25	0.026 (7)	0.040 (7)	0.029 (6)	−0.008 (5)	0.005 (5)	0.003 (5)
C26	0.019 (7)	0.032 (6)	0.031 (6)	−0.004 (5)	−0.006 (5)	−0.001 (5)
C27	0.015 (6)	0.025 (5)	0.032 (6)	−0.004 (4)	0.000 (5)	0.007 (4)

### Geometric parameters (Å, °)

**Table:**

Au1—Br1	2.4128 (13)	C10—C15	1.417 (16)
Au1—P1	2.246 (3)	C11—C12	1.400 (16)
Au1—Au2^i^	3.2574 (8)	C11—H11	0.9500
Au2—Br2	2.4170 (12)	C12—C13	1.389 (19)
Au2—P2	2.258 (3)	C12—H12	0.9500
Au2—Au1^ii^	3.2574 (8)	C13—C14	1.391 (19)
P1—C4	1.811 (10)	C13—H13	0.9500
P1—C1	1.815 (11)	C14—C15	1.377 (17)
P1—C10	1.822 (11)	C14—H14	0.9500
P2—C22	1.799 (12)	C15—H15	0.9500
P2—C16	1.826 (10)	C16—C21	1.388 (15)
P2—C3	1.829 (10)	C16—C17	1.419 (16)
C1—C2	1.540 (13)	C17—C18	1.395 (17)
C1—H1A	0.9900	C17—H17	0.9500
C1—H1B	0.9900	C18—C19	1.389 (17)
C2—C3	1.535 (14)	C18—H18	0.9500
C2—H2A	0.9900	C19—C20	1.397 (19)
C2—H2B	0.9900	C19—H19	0.9500
C3—H3A	0.9900	C20—C21	1.410 (16)
C3—H3B	0.9900	C20—H20	0.9500
C4—C5	1.413 (14)	C21—H21	0.9500
C4—C9	1.420 (15)	C22—C23	1.406 (16)
C5—C6	1.395 (15)	C22—C27	1.414 (16)
C5—H5	0.9500	C23—C24	1.379 (19)
C6—C7	1.375 (18)	C23—H23	0.9500
C6—H6	0.9500	C24—C25	1.36 (2)
C7—C8	1.426 (17)	C24—H24	0.9500
C7—H7	0.9500	C25—C26	1.378 (17)
C8—C9	1.387 (15)	C25—H25	0.9500
C8—H8	0.9500	C26—C27	1.380 (16)
C9—H9	0.9500	C26—H26	0.9500
C10—C11	1.417 (16)	C27—H27	0.9500
			
P1—Au1—Br1	171.73 (7)	C11—C10—C15	119.1 (10)
P1—Au1—Au2^i^	102.08 (7)	C11—C10—P1	120.7 (8)
Br1—Au1—Au2^i^	85.71 (3)	C15—C10—P1	120.0 (9)
P2—Au2—Br2	174.31 (8)	C12—C11—C10	119.0 (11)
P2—Au2—Au1^ii^	100.40 (7)	C12—C11—H11	120.5
Br2—Au2—Au1^ii^	84.87 (4)	C10—C11—H11	120.5
C4—P1—C1	106.3 (5)	C13—C12—C11	121.4 (12)
C4—P1—C10	104.5 (5)	C13—C12—H12	119.3
C1—P1—C10	103.1 (5)	C11—C12—H12	119.3
C4—P1—Au1	111.2 (4)	C14—C13—C12	119.1 (11)
C1—P1—Au1	116.3 (3)	C14—C13—H13	120.4
C10—P1—Au1	114.3 (4)	C12—C13—H13	120.4
C22—P2—C16	105.9 (5)	C15—C14—C13	121.4 (11)
C22—P2—C3	105.7 (5)	C15—C14—H14	119.3
C16—P2—C3	103.0 (5)	C13—C14—H14	119.3
C22—P2—Au2	114.6 (4)	C14—C15—C10	120.0 (11)
C16—P2—Au2	112.5 (4)	C14—C15—H15	120.0
C3—P2—Au2	114.1 (3)	C10—C15—H15	120.0
C2—C1—P1	114.1 (7)	C21—C16—C17	120.6 (10)
C2—C1—H1A	108.7	C21—C16—P2	119.5 (8)
P1—C1—H1A	108.7	C17—C16—P2	119.7 (8)
C2—C1—H1B	108.7	C18—C17—C16	118.7 (11)
P1—C1—H1B	108.7	C18—C17—H17	120.7
H1A—C1—H1B	107.6	C16—C17—H17	120.7
C3—C2—C1	111.3 (8)	C19—C18—C17	121.1 (12)
C3—C2—H2A	109.4	C19—C18—H18	119.5
C1—C2—H2A	109.4	C17—C18—H18	119.5
C3—C2—H2B	109.4	C18—C19—C20	120.0 (11)
C1—C2—H2B	109.4	C18—C19—H19	120.0
H2A—C2—H2B	108.0	C20—C19—H19	120.0
C2—C3—P2	112.7 (7)	C19—C20—C21	120.0 (10)
C2—C3—H3A	109.1	C19—C20—H20	120.0
P2—C3—H3A	109.1	C21—C20—H20	120.0
C2—C3—H3B	109.1	C16—C21—C20	119.6 (11)
P2—C3—H3B	109.1	C16—C21—H21	120.2
H3A—C3—H3B	107.8	C20—C21—H21	120.2
C5—C4—C9	118.7 (9)	C23—C22—C27	116.7 (11)
C5—C4—P1	119.3 (9)	C23—C22—P2	121.7 (10)
C9—C4—P1	121.7 (7)	C27—C22—P2	121.6 (8)
C6—C5—C4	120.2 (11)	C24—C23—C22	121.6 (13)
C6—C5—H5	119.9	C24—C23—H23	119.2
C4—C5—H5	119.9	C22—C23—H23	119.2
C7—C6—C5	120.9 (11)	C25—C24—C23	120.4 (12)
C7—C6—H6	119.5	C25—C24—H24	119.8
C5—C6—H6	119.5	C23—C24—H24	119.8
C6—C7—C8	119.9 (10)	C24—C25—C26	119.8 (12)
C6—C7—H7	120.0	C24—C25—H25	120.1
C8—C7—H7	120.0	C26—C25—H25	120.1
C9—C8—C7	119.6 (11)	C25—C26—C27	121.1 (12)
C9—C8—H8	120.2	C25—C26—H26	119.5
C7—C8—H8	120.2	C27—C26—H26	119.5
C8—C9—C4	120.5 (10)	C26—C27—C22	120.3 (10)
C8—C9—H9	119.7	C26—C27—H27	119.8
C4—C9—H9	119.7	C22—C27—H27	119.8
			
Br1—Au1—P1—C4	33.0 (7)	C1—P1—C10—C15	95.1 (9)
Au2^i^—Au1—P1—C4	−127.3 (4)	Au1—P1—C10—C15	−32.1 (10)
Br1—Au1—P1—C1	154.9 (6)	C15—C10—C11—C12	1.7 (16)
Au2^i^—Au1—P1—C1	−5.3 (4)	P1—C10—C11—C12	175.5 (9)
Br1—Au1—P1—C10	−85.0 (6)	C10—C11—C12—C13	−0.8 (17)
Au2^i^—Au1—P1—C10	114.7 (4)	C11—C12—C13—C14	−0.9 (18)
Br2—Au2—P2—C22	155.8 (7)	C12—C13—C14—C15	1.8 (18)
Au1^ii^—Au2—P2—C22	−46.6 (4)	C13—C14—C15—C10	−0.9 (18)
Br2—Au2—P2—C16	34.8 (9)	C11—C10—C15—C14	−0.8 (16)
Au1^ii^—Au2—P2—C16	−167.6 (4)	P1—C10—C15—C14	−174.7 (9)
Br2—Au2—P2—C3	−82.1 (9)	C22—P2—C16—C21	53.7 (10)
Au1^ii^—Au2—P2—C3	75.5 (4)	C3—P2—C16—C21	−57.1 (10)
C4—P1—C1—C2	−171.0 (7)	Au2—P2—C16—C21	179.6 (8)
C10—P1—C1—C2	−61.4 (9)	C22—P2—C16—C17	−130.5 (9)
Au1—P1—C1—C2	64.5 (8)	C3—P2—C16—C17	118.8 (9)
P1—C1—C2—C3	170.0 (7)	Au2—P2—C16—C17	−4.6 (10)
C1—C2—C3—P2	−81.0 (10)	C21—C16—C17—C18	−1.1 (17)
C22—P2—C3—C2	−176.9 (7)	P2—C16—C17—C18	−176.8 (9)
C16—P2—C3—C2	−65.9 (8)	C16—C17—C18—C19	1.8 (18)
Au2—P2—C3—C2	56.3 (8)	C17—C18—C19—C20	−1.7 (19)
C1—P1—C4—C5	−163.6 (9)	C18—C19—C20—C21	0.8 (19)
C10—P1—C4—C5	87.7 (9)	C17—C16—C21—C20	0.2 (17)
Au1—P1—C4—C5	−36.1 (10)	P2—C16—C21—C20	176.0 (9)
C1—P1—C4—C9	22.4 (11)	C19—C20—C21—C16	−0.1 (18)
C10—P1—C4—C9	−86.2 (10)	C16—P2—C22—C23	31.6 (12)
Au1—P1—C4—C9	150.0 (8)	C3—P2—C22—C23	140.5 (11)
C9—C4—C5—C6	1.1 (16)	Au2—P2—C22—C23	−93.0 (11)
P1—C4—C5—C6	−172.9 (9)	C16—P2—C22—C27	−149.1 (9)
C4—C5—C6—C7	0.6 (18)	C3—P2—C22—C27	−40.2 (10)
C5—C6—C7—C8	−2.5 (18)	Au2—P2—C22—C27	86.3 (9)
C6—C7—C8—C9	2.7 (18)	C27—C22—C23—C24	1(2)
C7—C8—C9—C4	−0.9 (17)	P2—C22—C23—C24	−179.9 (12)
C5—C4—C9—C8	−1.0 (17)	C22—C23—C24—C25	−2(2)
P1—C4—C9—C8	173.0 (9)	C23—C24—C25—C26	0(2)
C4—P1—C10—C11	32.3 (10)	C24—C25—C26—C27	1.9 (19)
C1—P1—C10—C11	−78.7 (9)	C25—C26—C27—C22	−2.7 (18)
Au1—P1—C10—C11	154.1 (7)	C23—C22—C27—C26	1.3 (17)
C4—P1—C10—C15	−153.9 (9)	P2—C22—C27—C26	−178.1 (9)

Symmetry codes: (i) −*x*+1/2, *y*−1/2, *z*; (ii) −*x*+1/2, *y*+1/2, *z*.
